# Emerging pharmacotherapies and regenerative solutions for promoting hair growth for androgenetic alopecia

**DOI:** 10.3389/fphar.2026.1776134

**Published:** 2026-03-16

**Authors:** Joshua Burshtein, Aaron Burshtein, Todd Schlesinger

**Affiliations:** 1 Department of Dermatology, University of Illinois Chicago, Chicago, IL, United States; 2 Icahn School of Medicine at Mount Sinai, New York, NY, United States; 3 Clinical Research Center of the Carolinas, Charleston, SC, United States

**Keywords:** alopecia, hair loss, hairregeneration, mechanism of action, novel therapies, traditional treatment

## Abstract

**Introduction:**

Androgenetic alopecia (AGA) is the most common form of non-scarring hair loss, characterized by progressive follicular miniaturization, shortened anagen phase, and increased telogen hairs. Traditional therapies, including topical minoxidil and oral finasteride, provide variable efficacy and often require long-term adherence, leaving a substantial unmet need. Emerging pharmacological and regenerative approaches aim to target underlying mechanisms of follicle degeneration, stimulate hair regrowth, and improve hair density and quality.

**Methods:**

A review of literature on novel treatments for AGA, focusing on pharmacological innovations and regenerative-medicine strategies, was performed. Original and review articles published before 1 December 2025 were evaluated for relevance.

**Discussion:**

Cell-derived exosomes and mesenchymal stromal cell–conditioned media have shown potential to activate dermal papilla cells, stimulate Wnt/β-catenin signaling, and enhance angiogenesis, resulting in improved hair density and shaft diameter in early clinical studies. Peptide-based formulations and growth-factor concentrates similarly demonstrate follicular anabolic effects, while PRP remains one of the most studied regenerative interventions, with evidence of additive benefit when combined with traditional therapies. Investigational pharmacotherapies, such as KX-826 (pyrilutamide), provide targeted androgen receptor modulation without systemic hormonal suppression. Despite promising preclinical and early clinical data, heterogeneity in study design, dosing, delivery methods, and follow-up limits direct comparisons and generalizability.

**Conclusion:**

Novel therapies for AGA offer biologically targeted, regenerative approaches that may complement or surpass traditional treatments. While preliminary evidence is encouraging, rigorous, standardized, and long-term clinical trials are required to establish efficacy, optimize protocols, and ensure safety. Future research integrating combination strategies and mechanistic insights is critical for translating these innovations into effective, durable treatments for patients with AGA.

## Introduction

Androgenetic alopecia (AGA) is the most common form of non-scarring hair loss, characterized by progressive miniaturization of hair follicles, shortening of the anagen phase, and a relative increase in telogen hairs (Kaiser et al., 2023; Pozo-Pérez et al., 2024; Krefft-Trzciniecka et al., 2023). Although traditional therapies such as topical minoxidil and oral finasteride have established efficacy, their effects are variable and they often require lifelong maintenance, which underscores a significant unmet need in clinical management (Kaiser et al., 2023; Pozo-Pérez et al., 2024). The psychosocial burden of AGA is substantial, with documented impacts on self-esteem, quality of life, and mental health, particularly when onset occurs at younger ages (Sawant et al., 2010).

In recent years, there has been an accelerating interest in novel pharmacological and regenerative-medicine approaches aimed at slowing hair-follicle miniaturization and stimulating new growth. Cell-derived exosomes from mesenchymal stem cells (MSCs) have been shown in preclinical and early clinical studies to activate dermal papilla cells via the Wnt/β-catenin pathway, thereby enhancing hair-growth signals (Fu et al., 2025). Similarly, stem-cell-based therapies have been reviewed in AGA, suggesting that autologous or allogeneic MSCs and follicle-derived stem cells may complement standard treatments (Krefft-Trzciniecka et al., 2023).

Additionally, pharmacotherapies, such as peptide-based formulations and growth-factor concentrates are emerging. A study of a biomimetic peptide solution reported improved hair density and shaft diameter in men with Norwood–Hamilton grade IV–VI AGA when used as adjunct to transplantation (Gold et al., 2025). Further, concentrated growth factor (CGF), a third-generation autologous platelet-derived concentrate, has been proposed as a scaffold for regenerative stimulation in AGA, with preliminary reports of improved hair density and favorable safety (Zhou et al., 2025).

This review aims to comprehensively summarize the latest innovations in the management of androgenetic alopecia, focusing on pharmacological advances and regenerative-medicine strategies. By evaluating recent human and translational studies, in this narrative review, we intend to outline the current state of the evidence, identify gaps in knowledge, and propose future directions for research and clinical application.

## Methods

A review of literature on novel treatments for AGA, focusing on pharmacological innovations and regenerative-medicine strategies, was performed. Databases searched include Pubmed, Embase, and ClinicalTrials.gov. Search terms used included “androgenetic alopecia treatment,” “alopecia,” “androgenetic alopecia treatment novel therapies,” and “hair regeneration.” Original articles, review articles, case reports/series published before 1 December 2025 were evaluated for relevance.

### Traditional management

The main pharmacological management of AGA involves the use of agents targeting chromosomal androgen-driven follicular miniaturization and/or stimulating hair-follicle cycling. The currently approved treatments by the Food and Drug Association (FDA) for AGA are topical minoxidil and oral finasteride (Rosenthal et al., 2024) (Table 1).

**TABLE 1:** Management of androgenetic alopecia.

Therapy	Mechanism of action	Clinical status (as of December 2025)	Regulatory approval
Topical minoxidil (2%–5%)	Potassium channel opener; prolongs anagen phase, promotes vasodilation and follicular blood flow	Well-established; multiple RCTs show increased hair density and count	FDA-approved (men and women)
Oral finasteride (1 mg)	Type II 5α-reductase inhibitor; reduces DHT production and androgen-driven miniaturization	Long-term data; increases hair density, stabilizes loss	FDA-approved (men only)
Oral dutasteride (0.5 mg)	Dual type I/II 5α-reductase inhibitor; more potent DHT reduction than finasteride	Superior efficacy vs. finasteride in meta-analyses; off-label use common	Not FDA-approved for AGA (approved for BPH); approved for AGA in Japan/South Korea
Low-dose oral minoxidil (off-label, e.g., 2.5–5 mg)	Same as topical: prolongs anagen, vasodilation; systemic delivery improves adherence	Growing off-label use; RCTs show comparable/superior efficacy to topical on vertex	Not FDA-approved for AGA (approved for hypertension)
Platelet-rich plasma (PRP)	Autologous growth factors (e.g., PDGF, VEGF); stimulates dermal papilla, reduces inflammation/fibrosis	Multiple RCTs/meta-analyses show increased density/thickness, especially + minoxidil	Off-label; no specific FDA approval for AGA
Concentrated growth factor (CGF)	Third-generation platelet concentrate; similar to PRP but higher growth factor release	Preliminary reports of improved density/safety	Off-label; no specific FDA approval for AGA
Exosome therapy (e.g., MSC/ADSC-derived)	Delivers bioactive molecules; activates Wnt/β-catenin, stimulates DPCs/HFSCs, promotes angiogenesis	Early clinical case series/small trials show density increases; heterogeneity in products/dosing	Off-label/experimental; no FDA-approved exosome products for any indication (including AGA)
Stem cell therapies (e.g., SVF, MSCs, conditioned media)	Regenerates HFSCs/DPCs; anti-inflammatory, paracrine signaling via growth factors/CM	Prospective/RCT data show hair count/density gains; meta-analyses support modest increases	Off-label/experimental; no FDA approval for AGA
Biomimetic/Growth-factor peptides (e.g., QR678 Neo, GPIGS, IGF-1 mimics)	Stimulate keratinocyte proliferation, VEGF production, hair shaft elongation/diameter	Small RCTs/*in vitro*/mouse models show promise; adjunct to transplantation	Off-label; no FDA approval for AGA
JAK pathway inhibitors (e.g., topical tofacitinib)	Reduce inflammation, increase VEGF; potential anagen restoration (limited AGA data)	Small studies/case reports; more evidence in alopecia areata	Off-label; no FDA approval for AGA
Pyrilutamide (KX-826)	Topical non-steroidal androgen receptor antagonist; blocks DHT at follicle without systemic DHT reduction	Mixed phase III results (some trials met safety/efficacy endpoints in China; prior failures noted); ongoing long-term/1.0% trials	Not FDA-approved; investigational (phase III ongoing/completed in parts)
VDPHL01 (extended-release oral minoxidil)	Extended-release formulation of minoxidil; sustained anagen prolongation/vasodilation	Phase 2/3 trials ongoing (enrollment complete in some male/female studies); promising early data vs. standard minoxidil	Not FDA-approved; investigational (phase 3 active, potential NDA ∼2027)

Topical minoxidil acts primarily by prolonging the anagen phase of the hair cycle and promoting follicular blood flow, thereby enhancing hair shaft thickness and density over time (Gupta et al., 2018). Numerous trials have demonstrated efficacy of topical minoxidil (Kaiser et al., 2023; Rosenthal et al., 2024; Gupta and Charrette, 2015). Topical minoxidil significantly increases total and non-vellus hair counts (Gupta and Charrette, 2015), while also improving hair density (Arca et al., 2004). Topical minoxidil requires twice-daily application, which may impact long-term adherence (Rosenthal et al., 2024).

Oral finasteride works through inhibition of type II 5-α reductase, which reduces conversion of testosterone into dihydrotestosterone (DHT). DHT is the androgen implicated in hair-follicle miniaturization (Kaufman et al., 1998). Clinical trials and long-term use show consistent increases in hair density, stabilization of hair loss progression, and improvements in hair appearance (Arca et al., 2004; Kaufman et al., 1998; Hu et al., 2015). In a large, randomized study of Chinese men, 80.5% of finasteride-treated men showed improvement after 12 months, compared with 59% in the 5% minoxidil group and 94.1% in the combined therapy group (Hu et al., 2015). Finasteride has reported adverse effects, including sexual side-effects such as decreased libido, erectile dysfunction, and ejaculatory disorders (Adil and Godwin, 2017; Mella et al., 2010). While the rate of these adverse effects is low around 2%–4% in males (McClellan and Markham, 1999; Mysore, 2012), this can be a concern of patients and may be a barrier to usage (Gupta and Charrette, 2014).

Since minoxidil and finasteride act via different pathways combining them has shown to yield additive benefits. A prospective randomized, assessor-blinded trial in men with AGA reported that combining 5% topical minoxidil plus 0.25% topical finasteride resulted in significantly greater increases in hair density at 3 and 6 months compared with either monotherapy (Rossi and Caro, 2024).

Dutasteride is another oral treatment option. It inhibits both type I and type II 5-alpha-reductase and is a more potent alternative to finasteride for treating AGA (Clark et al., 2004). Large, randomized controlled trials showed that dutasteride improved hair growth within 12–24 weeks and 6 months, respectively, compared with finasteride (Olsen et al., 2006; Eun et al., 2010). Comparing dutasteride to finasteride, recent meta-analysis showed that 0.5 mg dutasteride once daily was significantly more efficacious at increasing hair count compared with 1 mg finasteride once daily and 0.25/5 mg minoxidil once daily (Gupta et al., 2022). Since dutasteride has a partially similar mechanism of action to finasteride, there are reported related adverse effects. Head-to-head meta-analysis comparing dutasteride and finasteride found no significant difference for altered libido, erectile dysfunction, or ejaculation disorders over 24 weeks, suggesting comparable sexual-adverse-event profiles (Zhou et al., 2019).

Additionally, low-dose oral minoxidil has been shown to have beneficial efficacy from hair loss due to AGA (Gupta et al., 2024; Gupta et al., 2023a; Ong et al., 2025; Fazal et al., 2025; Chen et al., 2025; Akiska et al., 2025). While low-dose oral minoxidil is not FDA-approved for AGA, data suggests it improves hair density and terminal hair density, and is comparable in efficacy to topical minoxidil (Gupta et al., 2024; Penha et al., 2024). A recent randomized clinical trial showed that terminal hair density and total hair density was equivalent for the frontal and vertex areas (Penha et al., 2024). However, photographic analysis demonstrated oral minoxidil superior to topical minoxidil on the vertex but not on the frontal scalp (Penha et al., 2024). Low-dose oral minoxidil is a viable treatment option particularly for patients struggling with adherence or tolerability of topical minoxidil. It is a safe treatment with no significant effect on blood pressure (Chen et al., 2025).

In clinical practice, combining finasteride with low-dose oral or topical minoxidil is commonly implemented as a management strategy. A recent study with a combined oral minoxidil-finasteride regimen produced statistically significant and clinically meaningful improvement in hair density in AGA over 12 months (Johnson et al., 2025). Out of 502 men, 92.4% achieved stable or improved hair density, and 57.4% showed marked improvements (Johnson et al., 2025). In another study, at 12 months, 94.1% of men treated with oral finasteride plus topical 5% minoxidil showed improvement compared to 80.5% with finasteride and 59% with topical minoxidil alone (Hu et al., 2015). In addition, combined treatment with 5% topical minoxidil plus 0.25% topical finasteride significantly increased hair density over 6 months compared with either treatment alone (Rossi and Caro, 2024). The global photographic assessment score (GPAS) was also significantly higher compared to either treatment alone both at 3 and 6 months (Rossi and Caro, 2024).

However, despite the relative effectiveness of these standard therapies, limitations persist. The quality of evidence for many non-surgical treatments remains low or moderate, and variability among studies (in dosing, outcome measures, duration, and patient populations) complicates direct comparisons and generalizability (Gupta et al., 2018). The traditional standard-of-care for AGA has been centered on topical minoxidil and oral finasteride, alone or in combination, with growing use of off-label low-dose oral minoxidil. These therapies serve as a foundation for emerging pharmacological and regenerative strategies.

### Novel treatments

Advancements in the management of AGA have given rise to novel treatment strategies with the potential of improving care. The mechanisms of follicular miniaturization in AGA, i.e., hormonal, microenvironmental, and stem-cell exhaustion, are complex and not fully addressed by current therapies, leaving a substantial unmet need for more effective, durable, and biologically restorative treatments (Pozo-Pérez et al., 2024; McElwee and Sundberg, 2025). Over the past few years, advances in regenerative medicine, cell-based technologies, and targeted molecular therapies have opened new promising modalities, including exosome therapy, growth-factor or peptide formulations, stem-cell approaches, and platelet-rich plasma (PRP) (Table 1).

Several early clinical studies and systematic reviews describe that exosome-based therapies derived from mesenchymal stromal cells or adipose-derived stem cells (ADSC) can increase hair density and thickness in patients with AGA (Gupta et al., 2023b). Preclinical studies suggest that exosomes can improve hair growth by stimulating dermal papilla cells, activating hair follicle stem cells, and promoting angiogenesis (Gupta et al., 2023b; Queen and Avram, 2025). Clinical case series and small open-label trials have reported statistically significant increases in hair counts and patient-reported improvement after intradermal or topical application of ADSC-exosomes (Lee et al., 2024). A recent single-arm study using foreskin-derived MSC exosomes demonstrated improvements in vertex hair density and sustained patient satisfaction, although the level of evidence remains low and follow-up durations have been limited (Ersan et al., 2024). While exosomes have shown promise increasing hair growth through different mechanisms, i.e., activation of dermal papilla cells, stimulation of Wnt/β-catenin signaling, and enhanced angiogenesis, there is currently heterogeneity in manufacturing, dosing, and delivery methods across studies (Queen and Avram, 2025).

In addition to exosome therapy, biomimetic and growth-factor peptide formulations have shown improvements in hair growth. Topical or injectable peptide agents intend to stimulate follicular anabolism and improve shaft diameter in AGA (Gold et al., 2025; Kapoor et al., 2020a; Hwang et al., 2022; Hu et al., 2025; Iwabuchi et al., 2016). Randomized and comparative studies of multi-peptide formulations such as QR678/QR678-Neo report improvements in terminal hair counts, shaft diameter, and global photographic scores versus controls or PRP in some cohorts, though many trials are small and there is limited data (Kapoor et al., 2020b). Emerging procedural approaches combining peptides with microneedling or intradermal tattoo delivery (e.g., minoxidil-dutasteride-copper peptide tattooing) have shown promising early results in hair density, but these reports remain preliminary (Kuceki et al., 2025). Another study supported the use of a biomimetic peptide solution for rejuvenation of donor scalp and as storage media for hair follicle grafts during follicular unit extraction (FUE) hair transplantation surgery (Gold et al., 2025). They showed improvements in regrowth, density, and shaft diameter with the use of the peptide solution QR678 Neo (Gold et al., 2025). Several *in vitro* and mouse models have had positive results for novel peptide therapies. A topical penta-peptide agent, Gly-Pro-Ile-Gly-Ser (GPIGS), demonstrated proliferation of human hair keratinocytes and hair shaft elongation of human hair follicles in *vitro* models (Iwabuchi et al., 2016). A novel agent IGF-1 biomimetic peptide factor, Ac-GFFY-IGF, had superior efficacy to topical minoxidil, achieving faster hair regeneration, higher hair quality, and broader hair coverage at lower concentrations, in mouse models (Hu et al., 2025). Water-soluble egg yolk peptides have also been shown to stimulate VEGF production and human hair follicle dermal papilla cell growth in mouse models (Nakamura et al., 2018). While promising, biomimetic and growth-factor peptide formulations require additional investigation, with larger, controlled studies with long-term follow up to assess clinically meaningful effects.

Stem-cell based therapies show promise as well (Gasteratos et al., 2024). Both cellular (autologous MSCs or follicle-derived cells) and acellular (conditioned media, stromal vascular fraction, and exosome preparations) approaches have been evaluated for AGA (Krefft-Trzciniecka et al., 2023). They increasingly focused on using adipose-derived stromal vascular fraction (SVF), MSCs, or their conditioned media (CM) to stimulate hair regeneration beyond the capacity of traditional treatments. A prospective study in 30 patients with AGA demonstrated that a single injection of autologous SVF resulted in a significant increase in hair count density and hair shaft caliber (El-Khalawany et al., 2022). In a randomized, double-blind trial combining adipose-derived stem cell–conditioned media (ADSC-CM) with topical 5% minoxidil, treated areas showed significant increases in hair count, hair density, mean thickness, and terminal hair rate, along with a decrease in vellus hair proportion after 6 weeks of therapy (Legiawati et al., 2023). A meta-analysis further supports the potential of stem cell–derived CM. Among alopecia patients, including AGA, pooled analysis revealed a mean increase in hair density of ∼14.9 hairs/cm^2^ and a mean increase in hair thickness of ∼18.7 µm (Chien et al., 2024). These findings demonstrate that cell-based therapies, particularly SVF, MSCs, and CM/exosome approaches, may offer meaningful biologic regeneration in AGA beyond standard pharmacotherapy. Stem-cell based therapies remain an area of continued exploration and a potential future treatment for AGA with larger scale studies for further investigation.

PRP is among the most studied regenerative interventions for AGA. Studies show overall improvements in hair density, hair thickness, and anagen ratio versus baseline or placebo (Xiao et al., 2024). Pooled analysis demonstrated a significant increase in hair density (weighted mean difference [WMD] = 9.14; 95% confidence interval [CI]: 6.57-11.70) and hair diameter (WMD = 4.72; 95% CI 3.21-6.23) with PRP combined with minoxidil (Xiao et al., 2024). Patients receiving PRP with minoxidil also reported higher satisfaction rates compared to those using minoxidil or PRP alone (Xiao et al., 2024). Systematic reviews emphasize that PRP combined with minoxidil may yield additive benefit and that PRP protocols, i.e., centrifugation, platelet concentration, activation, injection interval strongly influence outcomes and interstudy heterogeneity (Evans et al., 2022). Randomized controlled split-scalp trials have demonstrated statistically significant increases in hair counts with PRP versus placebo (Shapiro et al., 2020). Hair density in PRP-treated areas increased from 151 ± 39.82 hairs/cm^2^ at baseline to 170.96 ± 37.14 hairs/cm^2^, a mean increase of approximately 20 hairs/cm^2^ (p < 0.05) (Shapiro et al., 2020). In addition, multiple randomized and controlled studies report significantly increased hair density and hair shaft thickness in AGA (Evans et al., 2022; Shapiro et al., 2020; Zhang et al., 2023). However, the extent of benefit varies across studies depending on the preparation method, treatment frequency, and patient characteristics. With numerous studies supporting the use of PRP paired with minoxidil, this is an area where traditional treatment can be enhanced.

There is limited evidence that JAK pathway inhibitors can be a treatment for AGA. Small studies, case reports and case series, of patients treated with oral JAK inhibitors for alopecia areata have occasionally manifested an AGA-pattern of remnant hair loss after regrowth, suggesting that JAK blockade can restore anagen but may not reverse established androgenetic miniaturization (Yale et al., 2020). In addition, topical tofacitinib, a JAK1 and JAK3 inhibitor, was found to promote hair growth more than minoxidil in C57BL/6 mice (p < 0.001). It was suspected that this occurred through increasing VEGF levels and reducing inflammation, suggesting that topical tofacitinib can be a potential treatment option for AGA (Meephansan et al., 2017). Further research into JAK pathway inhibitors is required to better characterize effect on hair growth and density for AGA, as current studies have small sample sizes and limited follow up data.

There is currently ongoing research into novel treatments for AGA. VDPHL01, a novel extended-release oral minoxidil is in clinical trials to assess efficacy (NLM, 2025; Veradermics, 2026). Preliminary results from a Phase 2 trial show that among 21 male participants who received VDPHL01 8.5 mg twice daily for 4 months, participants had an average increase in non-vellus hair count of 47.3 hairs/cm^257^. 90.5% of male patients receiving VDPHL01 reported seeing “improved” or “much improved” hair coverage, and 95% expressed increased satisfaction in their hair coverage (Veradermics). The open-label, multi-dose Phase 2 trial is still currently in progress as well as a multicenter, randomized Phase 3 clinical trial (Veradermics). This novel extended-release oral minoxidil has the potential to overcome the limitations of immediate-release minoxidil currently routinely used. Current oral minoxidil has a half-life of 3–4 h, while extended-release has the potential to remain in the system for longer periods of time and have a greater impact. Additional studies are needed for head-to-head efficacy comparisons once VDPHL01 is FDA approved (Akiska et al., 2025). In addition, KX-826, an investigational androgen receptor antagonist, is in phase 2 and 3 trials (To Evaluate Efficacy and Safety). It is also known as pyrilutamide, and works by blocking DHT’s effect on hair follicles without lowering overall DHT levels (To Evaluate Efficacy and Safety).

With evolving therapeutics, it is important to recognize that only topical minoxidil and oral finasteride are FDA approved for AGA. While topical minoxidil is FDA approved for both men and women, oral finasteride is approved only for men and not approved for women due to potential teratogenic effects on male fetuses. Dutasteride has been approved for AGA treatment in Japan and South Korea, but not in the. Treatments such as exosome therapy, growth-factor or peptide formulations, stem-cell treatments, and PRP are used off-label and do not have specific FDA-approval for AGA.

## Conclusion

The therapeutic landscape for AGA is rapidly evolving beyond traditional pharmacotherapies. Novel approaches, including regenerative-medicine strategies, cell-derived exosomes, stem-cell therapies, peptide and growth-factor formulations, and targeted molecular agents, hold significant promise for addressing the underlying mechanisms of follicular miniaturization and promoting durable hair regeneration. While early clinical and preclinical studies demonstrate encouraging improvements in hair density, thickness, and follicular function, substantial heterogeneity in study design, delivery methods, and outcome measures highlights the need for larger, standardized, and long-term clinical trials. Future research integrating combination therapies, optimized delivery systems, and mechanistic insights will be essential to translate these innovations into practical treatments for patients with AGA.
